# Metal-Coordinated Lignosulfonate Catalysts for the Selective Conversion of Hexose: Active Site and Reaction Medium

**DOI:** 10.3390/ma18245584

**Published:** 2025-12-12

**Authors:** Luyu Chen, Haoyu Zhang, Yirong Feng, Shuangfei Zhao, Lili Zhao, Wei He

**Affiliations:** 1 College of Biotechnology and Pharmaceutical Engineering, Nanjing Tech University, Nanjing 211816, China; 2 Institute of Advanced Synthesis, School of Chemistry and Molecular Engineering, Nanjing Tech University, Nanjing 211816, China; 3 State Key Laboratory of Materials-Oriented Chemical Engineering, Nanjing Tech University, Nanjing 210009, China

**Keywords:** lignosulfonat, acid site, glucose isomerization, hexose dehydration, 5-hydroxymethylfurfural

## Abstract

**Highlights:**

**What are the main findings?**

- Sustainable metal-coordinated lignosulfonate catalysts were selected as the model catalyst to reveal the role of active sites and solvents in hexose transformation.
- Lewis acid–base couple sites and Brønsted acid sites regulated the transformation process.

**What are the implications of the main finding?**

- Isomerization or dehydration pathway of glucose was adjustable via different solvents.
- Hf-LigS showed the best catalytic activity in hexose transformation, especially isomerization reaction at high glucose concentration in ethanol.

**Abstract:**

Sustainable lignosulfonate was employed as a key component for coordinating with various metal ions, affording metal-coordinated lignosulfonate catalysts (Hf-LigS) for hexose transformation. We first investigated the contribution of bare or tandem Lewis/Brønsted acid sites in each of the reaction steps. The results indicated that glucose isomerization was synergistically catalyzed by Lewis acid–base couple sites, and Brønsted acid sites were preferred for consecutive fructose dehydration to 5-hydroxymethylfurfural (HMF). DMSO and deep eutectic solvents enhanced the glucose dehydration and inhibited the isomerization reaction, leading to the formation of Levoglucosan (LGA) intermediate. Considering this, the Hf-LigS with moderate Lewis acid/base sites and Brønsted acid sites exhibited the best catalytic activity for hexose transformation, especially the isomerization reaction at high glucose concentration in ethanol (>20 wt%). This study established a fundamental understanding of the role of catalytic sites and solvent, guiding the selective transformation of hexose.

## 1. Introduction

Lignocellulosic biomass is the most abundant natural feedstock and has been employed as a potential replacement for fossil fuels [1,2]. The majority of lignocellulosic biomass consists of storage carbohydrates such as starch and cellulose, which are the most abundant in plants. These carbohydrates serve as the primary source of hexose sugars, including glucose and fructose. Currently, a variety of platform chemicals are produced from biomass via biorefining and biomanufacturing processes [3]. 5-Hydroxymethylfurfural (HMF) has been recognized as a pivotal C6 platform compound, which can be converted into a diverse array of valuable chemicals. Notably, HMF serves as a versatile precursor for valuable derivatives such as 2,5-dimethylfuran, a promising alternative to diesel fuel, and 2,5-furandicarboxylic acid (FDCA), which is a crucial building block for biopolymers [4,5,6]. Glucose generally undergoes a two-step transformation to yield HMF, including glucose isomerization to fructose and dehydration of fructose to HMF [7,8]. However, the mass production of HMF faces significant challenges, particularly in achieving high selectivity. Owing to its reactive functional groups, HMF is prone to side reactions, such as intermolecular condensation and rehydration, leading to the formation of byproducts including insoluble humins, levulinic acid, and formic acid. Consequently, the nature of catalytic sites has been identified as a crucial factor governing the HMF yield during hexose conversion [9,10,11]. Generally, in the process of glucose conversion to HMF, isomerization of glucose and fructose dehydration reaction were usually catalyzed by Lewis acid sites and Brønsted acid sites, respectively [12,13,14]. Ivanova et al. prepared a bifunctional activated carbon catalyst modified with toluenesulfonic acid and calcium to investigate the respective roles of Brønsted and Lewis acid sites in glucose hydrolysis. Their findings indicated that Lewis acid sites favored the isomerization pathway, whereas Brønsted acid sites directly catalyzed the dehydration of glucose to HMF via levoglucosan [15]. Sathitsuksanoh et al. developed phosphotungstic acid encapsulated MIL-101(Al)-NH_2_ for selective glucose conversion to HMF [16]. Their work demonstrated that a cooperative interplay and an optimal ratio between Lewis and Brønsted acid sites are crucial for achieving high HMF selectivity. Nevertheless, the majority of such studies have primarily examined the overall catalytic performance of bifunctional systems, without systematically disentangling the distinct roles and synergistic mechanisms of the individual acidic sites in complex reaction networks. A particular gap remains in the quantitative understanding of how tandem acid sites operate.

Complementary to catalyst design, changing the reaction medium could also adjust reaction rate, product selectivity, and reaction pathway [17]. Matubayasi et al. reported the solvent effect upon the reaction pathways and mechanisms for fructose conversion. They noted quite different pathways and rate constants [9]. Wu et al. also found that aprotic solvents could promote dehydration and inhibit isomerization reactions. In addition, to increase the yield of desired products and minimize the generation of unwanted condensation and polymerization products, a biphasic system with an extra organic phase to extract HMF from water has been proven effective [18]. Although the influence of the reaction medium is well acknowledged, studies that explicitly connect the design of catalyst active sites with solvent effects and proceed to systematically optimize both aspects are still limited.

The past few decades have witnessed the development of numerous heterogeneous acid–base catalysts, including transition-metal-containing zeolites, solid basic metal oxides, hydrotalcite, and niobium phosphate-supported magnesia tailored for enhanced activity, selectivity, and mechanistic control. Notably, metal–organic hybrid catalysts have attracted considerable attention due to their multiple synergistic active sites, which often lead to superior activity and selectivity compared to single-component catalysts. For example, significant efforts have been devoted to exploring metal–organic frameworks (MOFs) for hexose conversion, primarily through functionalized MOF structures and MOF-zeolite composites [19,20]. More importantly, the diversity of organic ligands could endow catalysts with designability and multifunctionality. It was reported that Al3+ ions were not recognized as effective photocatalysts to achieve photocatalytic conversion of sugars to HMF. However, complexing them with fulvic acid components as light antennas created new functionality [21]. However, fundamentally, systematic experimental evidence and a deep understanding of the specific regulatory roles of isolated versus tandem Lewis/Brønsted acid sites in hybrid catalysts remain lacking. It is known that the type and strength of active sites in hybrid catalysts can be readily modulated based on their distinct functional groups, offering opportunities for precise site design and structure–activity relationship investigations in this study.

Sulfonic acid resins, known for their strong acidity and high catalytic activity, can provide a stable acidic environment for reactions, making them widely employed to enhance the dehydration rate. As the second most abundant component of lignocellulosic biomass, lignosulfonate (LigS), derived from the pulp and paper industry, has found broad application as a water reducer and oilfield chemical additive. The stable structure, high thermal stability, and inherent sulfonic groups in LigS offer a foundation for designing Brønsted acid catalysts based on this biomass material. Additionally, the coordination between metal elements and phenolic hydroxyl groups in LigS can generate Lewis acid–base active sites. Therefore, LigS and its derivatives show potential as multifunctional catalysts, analogous to sulfonic acid resin systems. Recently, LigS catalysts have been developed as novel solid acids in catalysis fields, including reductive upgrading of HMF [22], Meerwein–Ponndorf–Verley (MPV) reaction of aldehydes [23], transferhydrogenation of levulinic acid [24], providing a novel approach for turning waste into wealth. Sn-*β* catalysts have also been employed for glucose conversion. For instance, Holm et al. documented that the Sn-*β* catalyst facilitated glucose isomerization, resulting in a 41.5% fructose yield. By incorporating an acidic resin in a one-pot system, they further achieved a 56% furfural yield from glucose conversion [25]. In another study, Gong et al. demonstrated that under microwave-assisted conditions, the UiO-66 catalyst reacted with glucose in dimethylsulfoxide at 160 °C for 30 min, dehydrating it to produce HMF with a maximum yield of 37%. Under identical conditions, the MOF-808 catalyst achieved an optimal HMF yield of 31% [26]. However, the current literature provides limited insights into the relationship between the structural features of active sites and catalytic efficiency. A notable research gap remains in understanding how to precisely design and differentiate isolated versus synergistic acid sites based on the molecular architecture of lignosulfonate (LigS), and further, how to quantitatively evaluate their catalytic performance across varying reaction environments.

In response to these research gaps, we developed metal-coordinated lignosulfonate catalysts for hexose conversion. Our key innovation lies in precisely decoupling the catalytic roles of different acid sites through rationally designed monofunctional catalysts. This approach uniquely quantifies the distinct contributions of isolated versus tandem Lewis/Brønsted acid sites, a crucial aspect previously overlooked. Comprehensive characterization, systematic solvent screening, and kinetic studies collectively establish a clear structure–activity relationship, providing unprecedented insights into the reaction mechanism.

## 2. Materials and Methods

Sodium lignosulfonate (96%), alkali lignin (EP grade), and levulinic acid (99%) were purchased from Shanghai Macklin Biochemical Technology Co., Ltd. (Shanghai, China). Glucose (AR), fructose (AR), chromium (IV) chloride (98%), zirconium (IV) chloride (98%), and choline chloride (98%) were purchased from Shanghai Aladdin Biochemical Technology Co., Ltd. (Shanghai, China). Ruthenium (III) chloride (99%) was purchased from Beijing J&K Scientific Co., Ltd. (Beijing, China). Iron (III) chloride (98%) was purchased from Alfa Aesar (Shanghai, China) Chemical Co., Ltd. (Shanghai, China). Concentrated sulfuric acid (98%) and dimethyl sulfoxide (DMSO, AR) were purchased from Shanghai Lingfeng Chemical Reagent Co., Ltd. (Shanghai, China). 5-Hydroxymethylfurfural (HMF, 99%), formic acid (98%), absolute ethanol (AR), and sodium chloride (99%) were purchased from Sinopharm Chemical Reagent Co., Ltd. (Shanghai, China). Sorbitol (98%) was purchased from Adamas Reagent Co., Ltd. (Shanghai, China). Levoglucosan (1,6-anhydro-β-D-glucopyranose, 99%) was purchased from Shanghai Yuan’en Technology Development Co., Ltd. (Shanghai, China). D-Glucuronic acid (98%) was purchased from Shanghai Yuanye Bio-Technology Co., Ltd. (Shanghai, China). Mannose (99%) was purchased from Shanghai Aladdin Biochemical Technology Co., Ltd. (Shanghai, China).

Analyses were performed using the following instruments: an analytical balance (model ME204, Mettler Toledo International Trading Co., Ltd., Shanghai, China); a magnetic heating stirrer (model 2KA-RCT digital, IKA (Guangzhou) Instruments & Equipment Co., Ltd., Guangzhou, China); a high-speed centrifuge (model H1650, Shanghai Xiangfan Instrument Co., Ltd., Shanghai, China); an electro-thermostatic blast drying oven and a vacuum drying oven (models 101-1AB and DZ-2BCIV, respectively, Tianjin Test Instrument Co., Ltd., Tianjin, China); an ultrasonic cleaner (model SK5200BT, Shanghai Kedao Ultrasonic Instrument Co., Ltd., Shanghai, China); an Agilent high-performance liquid chromatography (HPLC) system (model 1260, Agilent Technologies (China) Co., Ltd., Shanghai, China); and a PURELAB water purification system (Veolia Water Technologies China Co., Ltd., Shanghai, China). Data graphs were generated using Origin 2018 software (64-bit, OriginLab Corporation, Northampton, MA, USA).

### 2.1. Catalysts Preparation

The inorganic–biopolymer hybrids (Hf-LigS) catalysts were synthesized by a facile hydrothermal process. HfCl_4_ and LigS in the same mass were dissolved in deionized water, respectively. Then the corresponding aqueous solution was placed in a 50 mL thick-walled pressure tube. The coordination reaction was operated at 120 °C for 12 h. After being cooled, the resulting mixtures were centrifuged to yield a powder. The resulting powder was washed with deionized water and ethanol, affording catalysts after vacuum drying at 60 °C for 12 h. As a comparison, sodium lignosulphonate was replaced by alkali lignin and FDCA, obtaining Hf-Lig and Hf-FDCA hybrid catalysts. LigS was dissolved in deionized water, and then concentrated sulfuric acid was added while stirring until precipitation was formed. After the reaction was operated at room temperature for 30 min, the solid precipitate was filtered and washed with deionized water and ethanol. Then, it was dried in a 60 °C oven to obtain the Acid-LigS catalyst. Finally, a series of characterizations was conducted to obtain the structure, morphology, composition, and active sites of the resulting catalysts.

### 2.2. Catalytic Reaction of Hexose

Reactions for glucose isomerization and hexose (glucose or fructose) dehydration to HMF were carried out in a 35 mL thick-walled pressure tube. The function of catalytic sites was probed in ethanol, employing Hf-LigS as the catalyst, which was also used to evaluate solvent effects. Unless otherwise specified, the experiments were operated by following these steps. After the reaction, the solid catalyst was recovered by centrifugation, washed thoroughly with the reaction solvent and deionized water, and then dried under vacuum at 60 °C for reuse. Additionally, the resulting liquid phase was directly analyzed by HPLC to determine the product yield and distribution.

#### Glucose Isomerization Reaction

In a standard assay, 5 mg of glucose and 5 mg of catalyst (Hf-LigS, Hf-Lig, or Acid-LigS) were dispersed in 1 mL of ethanol and reacted at 100 °C for a set time. The spent catalyst was then separated for recycling. The liquid phase was quenched with 5 mM H_2_SO_4_ for one hour at room temperature and then analyzed by HPLC. Any incomplete carbon balance was accounted for by humin formation. To evaluate solvent effects, ethanol was substituted with other media, including water, tetrahydrofuran (THF), acetonitrile, methanol, and isopropanol.

### 2.3. Fructose Dehydration Reaction

The roles of solvent and active sites were evaluated by performing fructose dehydration in DMSO, ethanol, and DESs using Hf-LigS, Hf-Lig, and Acid-LigS catalysts. Specifically, the reaction in DMSO involved 30 mg fructose, 4 mg catalyst, 0.2 mL saturated NaCl, and 1 mL DMSO, stirred at 140 °C for 1 h. In parallel, the ethanol system utilized 30 mg fructose, 7 mg catalyst, 0.9 mL saturated NaCl, and 0.9 mL ethanol, reacted under the same temperature and duration. For DESs, a mixture of 20 mg fructose and 30 mg choline chloride was first prepared, followed by the addition of 7 mg catalyst and reaction at 120 °C for 1 h. In situ NMR monitoring was performed to track the reaction progress in real time. A mixture of fructose (30 mg), Hf-LigS catalyst (20 mg), and DES/DMSO-d6 (0.5 mL) was loaded into an NMR tube and heated at 120 °C. ^1^H and ^13^C NMR spectra were acquired continuously throughout the reaction.

### 2.4. Direct Conversion of Glucose into HMF

The glucose dehydration reactions were conducted by the following steps with different solvent media. With DMSO or ethanol as the solvent, 20 mg of glucose, 7 mg of catalyst (Hf-LigS or Hf-Lig or Acid-LigS), 0.6 mL of saturated sodium chloride solution, and 2.4 mL of solvent (DMSO or ethanol) were added to the tube, and the reaction occurred at 170 °C for 3 h. With DES as the solvent, glucose (20 mg), choline chloride (30 mg), and 7 mg of catalysts were mixed in the tube, and the solution was stirred and reacted at 130 °C for 3 h.

## 3. Results and Discussion

### 3.1. Catalytic Characterization

**Figure 1:**
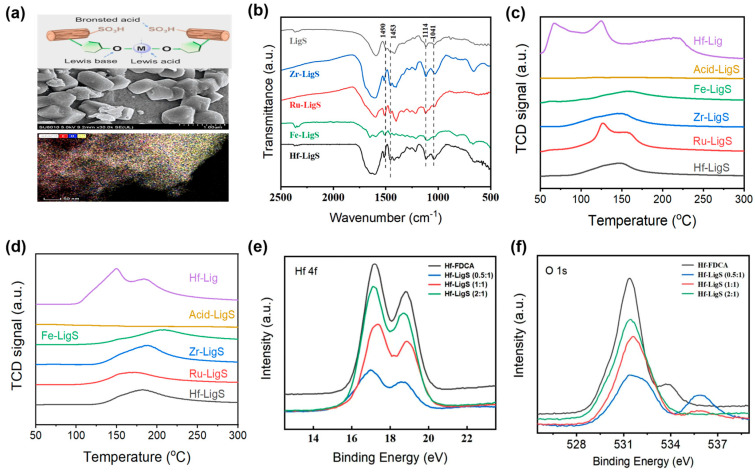
(**a**) Plausible structure of acid–base Hf-LigS, SEM and HAADF-STEM images of Hf-LigS; (**b**) FT-IR spectra; (**c**) NH_3_-TPD curves; and (**d**) CO_2_-TPD curves of Hf-LigS, Hf-Lig, and Acid-LigS. High-resolution XPS spectra of Hf-LigS and Hf-FDCA: (**e**) Hf 4f and (**f**) O 1s.

Figure 1a illustrates the proposed structure of the acid-base bifunctional metal–lignosulfonate hybrid. Representative characterization of Hf-LigS by SEM and TEM (Figure 1a and Figure S1) identified an amorphous and blocky morphology, while elemental mapping evidenced the homogeneous distribution of C, O, S, and Hf. Comparative FT-IR analysis (Figure 1b) of LigS and Hf-LigS further revealed structural modifications resulting from the coordination process. Infrared spectroscopic analysis further confirmed the structural integrity of the Hf-LigS catalyst after reaction. As shown in Figure S1, the characteristic absorption peaks corresponding to the -SO_3_H groups remained clearly observable, indicating that these functional groups remained intact under the applied reaction conditions. The peaks of LigS at 1490 cm^−1^ and 1453 cm^−1^ correspond to anti-symmetric -Ar-OH stretching and symmetric -Ar-OH stretching of the phenolic hydroxyl groups from the lignosulfonate chains [22]. It was found that the bands of anti-symmetric -Ar-OH stretching from Hf-LigS were shifted to a higher frequency compared with that of LigS, which confirmed the coordination of phenolic hydroxyl groups and metal ions. Moreover, the characteristic peaks at 1114 cm^−1^ and 1041 cm^−1^ indicated the remained sulfonic groups after the hydrothermal process [27,28,29]. As demonstrated in TGA curves (Figure S2), the thermal stability of Hf-LigS was improved because of the strong interactions between Hf^4+^ and phenolic hydroxyl groups compared with LigS.

Subsequently, the features of acidic/basic active sites for those catalysts were explored by NH_3_-TPD and CO_2_-TPD methods. A larger amount of NH_3_ and CO_2_ desorbed, and a higher desorption temperature indicated a higher content and strength of acidic/basic species in the catalysts. The NH_3_-TPD spectra (Figure 1c) showed that desorption peaks of Hf-LigS were below 200 °C, which demonstrated that Hf-LigS catalysts only had weak acid sites. The XPS binding energy shift indicated the relative Lewis acid strength within the series of synthesized Hf-LigS catalysts, with the 1:1 ratio being the strongest. In contrast, the NH_3_-TPD profile, showing desorption peaks below 200 °C, classified the absolute acid strength of Hf-LigS as weak compared to general acidic materials. The nature and content of acid sites in Hf-LigS were also analyzed by pyridine-FTIR characterization (Figure S3), indicating the existence of Lewis acid sites and Brønsted acid sites [22]. The Lewis acid site content (0.058 mmol/g) in Hf-LigS was higher than that of Brønsted sites (0.019 mmol/g) at 110 °C, consistent with its Hf-rich composition. Notably, Hf-Lig displayed stronger acidity, as evidenced by three NH_3_-TPD peaks-two below 200 °C and one in the 200–400 °C range. Quantitative analysis (Table S1 confirmed the acidity trend: Hf-Lig > Hf-LigS > Acid-LigS. Moreover, CO_2_-TPD spectra showed that Hf-LigS had a weak desorption peak below 200 °C (Figure 1d). Acid-LigS had no desorption peak, and Hf-Lig had two obvious weak desorption peaks below 200 °C. The basicity of these catalysts was as follows: Hf-Lig > Hf-LigS. The corresponding acid/base ratio, in descending order, was Hf-Lig > Hf-LigS. Therefore, the coordination of metal chloride (metal-centers with Lewis acid characteristics) and LigS (O-centers with Lewis base characteristics) endowed Hf-LigS hybrids with Lewis acid and base sites.

Catalysts with different Hf contents were obtained by adjusting the feed mass ratio of HfCl_4_ to LigS. In ICP-OES detection, when the feed mass ratios were 0.5:1, 1:1, and 2:1, the mass fractions of Hf in catalysts were 25.2%, 36.6%, and 49.9%, respectively. Figure 1e presents the characteristic energy spectrum of Hf 4f. In spectra of HfO_2_, the binding energies at 16.45 eV and 18.02 eV were assigned to Hf 4f 7/2 and 4f 5/2 [30]. When the Hf atom was coordinated with the O atom in the phenolic hydroxyl group of LigS, the difference in electronegativity between O and the Hf atom would drive the electrons to transfer from Hf to O, resulting in a more positive charge in the Hf atom and a higher binding energy of Hf 4f [31]. Among the Hf-LigS hybrids with varying mass ratios, the 1:1 sample showed the highest Hf 4f binding energy, suggestive of the strongest Lewis acidity. This enhanced acidity was further evidenced when compared to Hf-FDCA (1:1), with Hf-LigS (1:1) displaying a superior Hf 4f binding energy. The characteristic O 1s spectrum is shown in Figure 1f. The binding energy of O1s in Hf-containing hybrids fell between 530.5 eV (HfO_2_) and 532.94 eV (sulfonic acid group in LigS) [32,33], which indicated that the functional groups in LigS could stabilize Lewis acidic Hf^4+^ species and increase the content of basic species by the formation of -Ar-O-Hf-O-Ar- framework structure.

### 3.2. Effect of Catalytic Active Site on Catalytic Performance

Three hexose conversion processes, including glucose isomerization, glucose dehydration, and fructose dehydration, were chosen to explore the influence of catalytically active sites in Hf-LigS catalysts on their catalytic performance.

#### 3.2.1. Glucose-to-Fructose Isomerization

**Figure 2:**
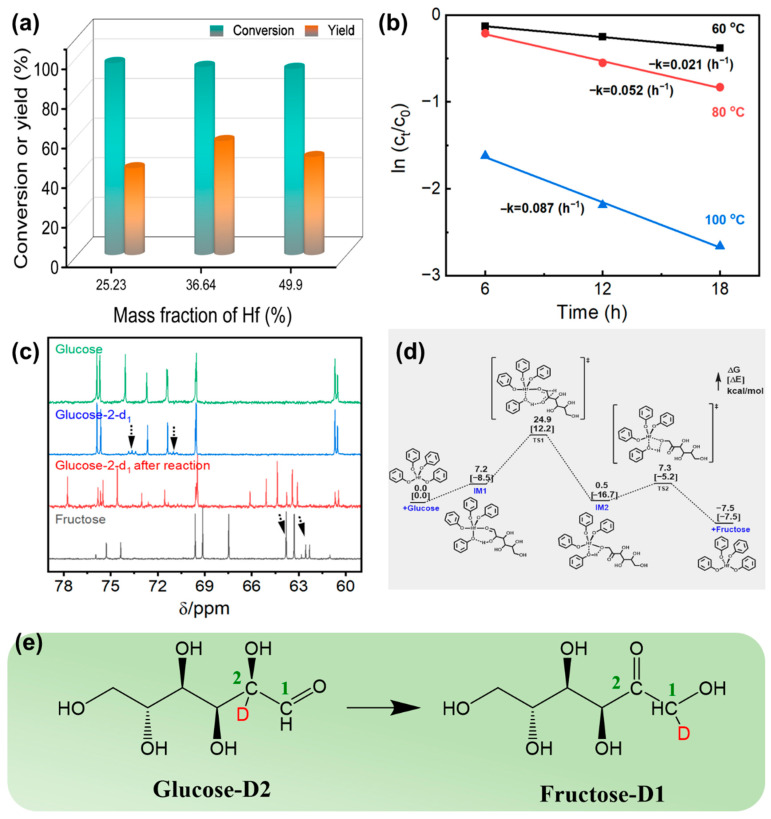
(**a**) Effect of Hf content on the glucose conversion and fructose yield, time = 18 h. (**b**) Linear fitting of ln k vs. 1000/T. (**c**) ^13^C NMR spectra of glucose-2-d1 before and after the reaction in ethanol. (**d**) Mechanism diagram of Lewis acid–base site-catalyzed glucose isomerization. Here, the steps marked with “≠” in the figure represent irreversible reactions. (**e**) Intramolecular hydride shift mechanism.

At first, the catalytic performance was evaluated in the isomerization of glucose (Figure 2a). Hf-LigS displayed the highest fructose yield (40.7%) with high glucose conversion (77.4%). When LigS was used as the catalyst, the conversion and selectivity were 79.9% and 36.9%, respectively. In addition, Hf-Lig afforded a conversion of 65.5% and a selectivity of 32.3%. The effect of Hf contents of Hf-LigS on catalytic performance was also investigated. When the Hf content was 36.6%, the highest binding energy of Hf 4f was observed (Figure 1e), and the catalytic activity of Hf-LigS was the best (Figure 2b), affording fructose with a yield of 57.8%. The catalytic performance of Hf-LigS was highly dependent on its Hf content, with a moderate acid site density proving optimal for glucose isomerization. Deviations from this optimum, in either direction, led to suboptimal fructose yields. Furthermore, the Hf-FDCA hybrid, possessing coexisting Lewis acid and base sites, demonstrated considerably lower activity and selectivity compared to Hf-LigS, highlighting its inferior performance. These control experiments demonstrated a positive cooperative effect between HF^4+^ and sodium lignin sulfonate on the isomerization reaction. The effect of different active sites on the product distribution and reaction kinetic constants was included in the glucose dehydration section.

Generally, the fructose yield reaches over 50.0% at low glucose concentration (no more than 10 wt%) in ethanol [34]. We explored the performance of glucose isomerization under different initial glucose concentrations. As shown in Figure S4, a high yield (58.4%) for fructose was detected when the glucose loading was increased to 21 wt%. Hf-LigS exhibited high production capabilities at high feed concentrations, which were higher than those of hydrotalcite catalyst, zeolites, and MgO/nitrogen-doped carbon materials [34,35,36,37]. The reaction kinetics of glucose isomerization in the presence of Hf-LigS were studied. As depicted in Figure 2c and Figure S6, the plot of lnk against 1/T gave a straight line, exhibiting a typical Arrhenius behavior. The apparent activation energy (Ea) was calculated to be 8.84 kcal/mol at a low conversion rate (below 20.0%). A lower Ea was observed than that of the previously reported base-catalyzed isomerization process [38].

The mechanism was investigated through isotopic labeling experiments with deuterium-labelled glucose (glucose-2-d1) as the substrate. In the ^13^C NMR spectra of glucose-2-d1 (Figure 2e), the resonance peaks at *δ* = 74.1 ppm and 71.3 ppm showed as low-intensity triplets, which were ascribed to the disruption of the nuclear Overhauser enhancement (NOE) by the deuterium atoms in the C-2 positions of the two configurations of glucose-2-d1 in solution (*β*-pyranose and *α*-pyranose) [39]. After reaction, the products had differences compared to the unlabeled fructose. The peaks at *δ* = 63.8 and 62.6 ppm belonged to the C-1position of *β*-pyranose and *β*-furanose configurations in the unlabeled fructose. However, these resonances appeared as low-intensity peaks in the solution after reacting labeled glucose-2-d1, which indicated that the deuterium atom located in the C-2 position of glucose-2-d1 has moved to the C-1 position of fructose. In the 1H NMR spectra after reaction (Figure S5), the disappearance of the resonance at *δ* = 3.45 ppm, because of the deuterium atom in the C-1 position further confirms these results. This observation corroborated that the isomerization of glucose with Hf-LigS catalyst operated in the form of an intramolecular hydride shift (Figure 2d), similar to that in the presence of a Lewis acid catalyst.

Density functional theory (DFT) calculations were employed to probe the synergy of Lewis acid–base sites in Hf-LigS (computational details in Table S3 and Figure S12). The free energy profile (Figure 2e) showed that the reaction initiates when the glucose aldehyde oxygen bonds to the Hf center, thereby forming the initial complex IM1. The complex IM1 was energetically more stable (i.e., ∆E = 8.5 kcal/mol) but less stable in free energy (i.e., ∆G = 7.2 kcal/mol) than the separated catalyst and glucose, which implied that the electrostatic attraction between Hf- and O-center and weak OH—O hydrogen binding interaction could benefit the attack by pulling the Hf-catalyst and glucose together. After crossing a barrier (TS1) of 17.7 kcal/mol, the H-transfer from the HO-group at the C*α* position of glucose to one PhO-ligand of the catalyst, together with H-transfer from C*α*-H to the terminal aldehyde group, was concurrently achieved, thus generating the intermediate IM2, in which the carbonyl group was formed at the C*α* position of glucose species. The glucose isomerization proceeds via a synergistic mechanism involving the Lewis acid Hf center and the basic PhO-ligand. The overall barrier was calculated as 24.9 kcal/mol, amenable to mild conditions. The slightly endothermic (+0.5 kcal/mol) formation of IM2 suggests a reversible hydrogen transfer/carbonyl formation step. This is followed by a facile H-atom transfer via a four-membered-ring transition state (TS2) with a barrier of 6.8 kcal/mol. These findings demonstrate that the Lewis acid-base synergy in Hf-LigS lowers the activation energy, enabling efficient glucose isomerization.

#### 3.2.2. Fructose Dehydration to HMF

**Figure 3:**
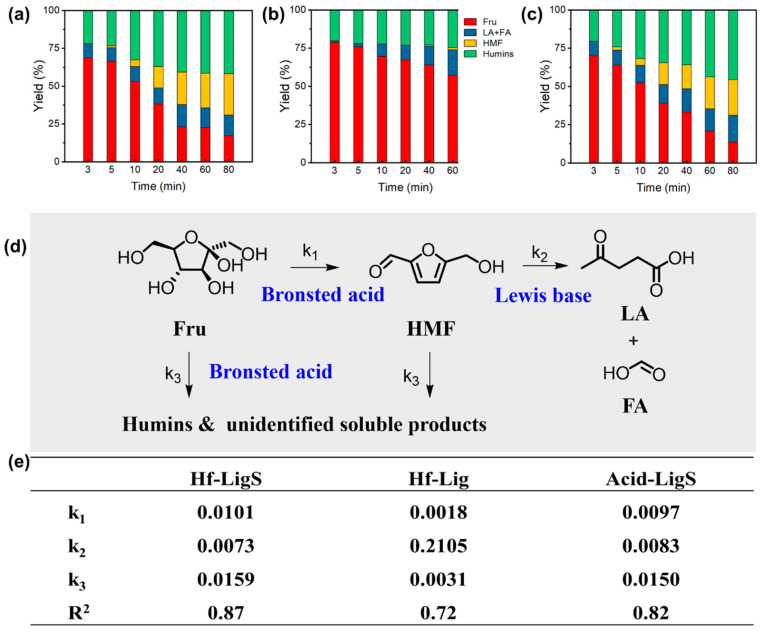
Product yield distribution over Hf-LigS (**a**), Hf-Lig (**b**), and Acid-LigS (**c**). (**d**) Catalytic constant of each reaction step. (**e**) Lewis/Brønsted acid sites’ role in fructose dehydration.

In the fructose dehydration reaction, Hf-LigS exhibited the best catalytic performance (Figure 3a), achieving a fructose conversion of 80.5% and an HMF yield of 26.4%. To elucidate the individual and combined roles of Lewis and Brønsted acid sites, control experiments were conducted using Hf-Lig (Lewis acid–base sites), Hf-LigS (Lewis acid–base and Brønsted acid sites), and Acid-LigS (solely Brønsted acid sites). As shown in Figure 3b–d, fructose conversion increased with reaction time in all systems. However, Hf-Lig showed the lowest catalytic efficiency and primarily produced formic acid (FA) and levulinic acid (LA) as the main products. In contrast, both Acid-LigS and Hf-LigS displayed similar product distribution patterns, with the HMF yield rising rapidly over time. These results further confirm that Brønsted acid sites exhibit significantly higher activity than Lewis acid–base sites in promoting fructose dehydration to HMF.

To quantify the influence of different acid sites, a kinetic model of the fructose dehydration network was established (Figure S7), enabling the determination of individual rate constants. The derived rate equations, expressed in terms of component concentrations (Fru, HMF, humin, LA, FA) and rate constants (k_1_–k_3_), closely matched the experimental trends (Figure S7). Analysis of the constants (Figure 3e) showed that Hf-LigS and Acid-LigS, both possessing Brønsted acid sites, afforded similar k_1_–k_3_ values. Their notably high k_1_ value highlights the essential function of Brønsted acid sites in the initial dehydration step to form HMF. Meanwhile, the significantly elevated k_2_ value for Hf-Lig verifies that Lewis base sites enhance HMF hydration, while its minimal k_3_ value suggests that Brønsted acidity is a key factor in humin generation.

#### 3.2.3. Glucose Dehydration to HMF

**Figure 4:**
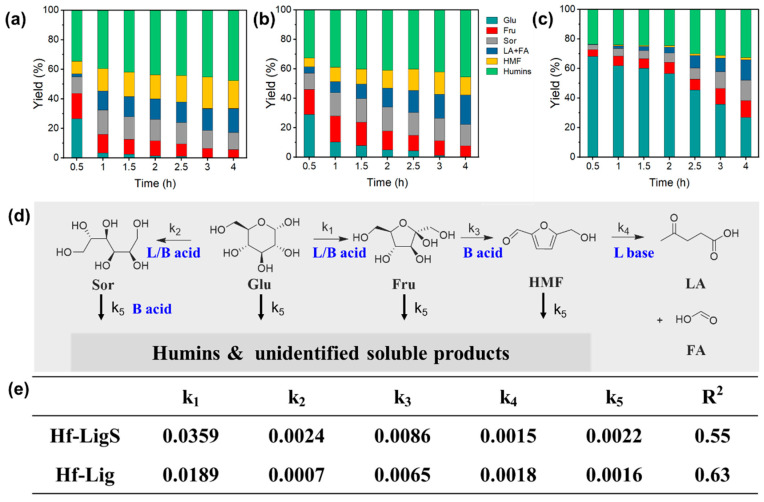
Product yield distribution over (**a**) Hf-LigS, (**b**) Hf-Lig, and Acid-LigS (**c**). Main reactions involved in the kinetic model (**d**) and catalytic constant of each reaction step (**e**).

The conversion of glucose to HMF through a one-pot method was also investigated (Figure 4a). Using Hf-LigS, nearly full glucose conversion was attained with an HMF yield of 20.7%. Investigations into the role of acid–base sites (Figure 4b–d) revealed that glucose was rapidly consumed within 1 h over Hf-Lig and Hf-LigS. In marked contrast, reactions with Acid-LigS left 25% glucose unreacted after 4 h and produced negligible HMF, indicating that the isomerization step is hindered without Lewis acid–base sites. The higher HMF yield from Hf-LigS versus Hf-Lig demonstrates the synergy of combined Lewis acid–base and Brønsted acid sites in the dehydration cascade. The product distribution analysis further reveals the reaction pathway selectivity. We clarify that while the primary pathway in ethanol is glucose isomerization to fructose (due to the Lewis acid–base sites), a minor amount of sorbitol is detected, indicating a competing reduction pathway. However, the kinetic constants (k_1_ for isomerization vs. k_2_ for sorbitol formation) confirmed that isomerization was the significantly faster process under the catalytic conditions.

Based on the literature report [40], a multi-step reaction model and the calculation formula for the reaction constant were constructed (Figure 4e and Figure S8). Firstly, glucose could be converted to fructose or sorbitol. Fructose was further dehydrated to HMF, and HMF would undergo a hydration reaction to generate LA and FA. All the substrates and intermediates in the above reactions could convert to humin through further polymerization reactions. The parameters of the kinetic model were obtained (Figure 4e).

A kinetic comparison between Hf-LigS and Hf-Lig selected due to the inactivity of Acid-LigS in Lewis acid/base catalysis revealed that Hf-LigS exhibits twice the isomerization rate constant (k_1_) and a significantly higher sorbitol formation rate. The overall HMF formation was found to be limited by the fructose dehydration step. These results, supported by control experiments and kinetic modeling, indicate that glucose isomerization is selectively promoted by the synergy of Lewis acid/base and Brønsted acid sites. Maximizing fructose yield requires moderate Lewis acidity to suppress side reactions, while subsequent fructose dehydration is favored by Brønsted acidity, though excessive strength leads to humin formation. Thus, an optimal catalyst for HMF should feature balanced Lewis acid–base and moderate Brønsted acid sites.

### 3.3. Effect of Reaction Medium on Catalytic Performance

In addition to active sites, the reaction solvent was another crucial consideration for the transformation of glucose [9,10]. Thus, the solvent effect was also investigated in the presence of the Hf-LigS catalyst.

#### 3.3.1. Glucose-to-Fructose Isomerization

**Figure 5:**
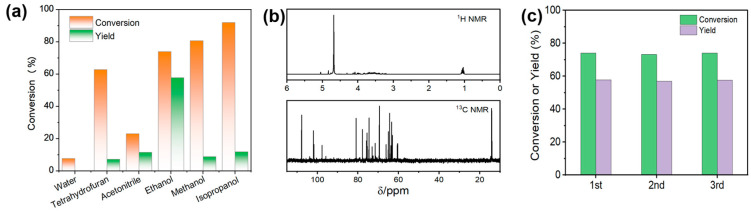
(**a**) Effect of different solvents on glucose isomerization, time = 18 h. (**b**) ^1^H NMR and ^13^C NMR spectra of products in ethanol. (**c**) Reusability of Hf-LigS, time = 18 h.

This isomerization over Hf-LigS was operated in different solvents. The isomerization reaction in tetrahydrofuran, acetonitrile, and water yielded less fructose. Compared with methanol and isopropanol, ethanol was the best solvent for the isomerization reaction, with the glucose conversion of 74.0% and a yield of fructose up to 57.8% (Figure 5a). The products in ethanol were identified by ^13^C and ^1^H NMR spectroscopy. The resonances *δ* = 65.0 and 14.1 ppm in the ^13^C NMR spectra, as well as *δ* = 1.2 ppm in the 1H NMR spectra (Figure 5b), demonstrated the formation of ethyl fructoside that shifted the equilibrium towards the side of product generation [41,42]. Subsequently, the ethyl fructoside intermediate can be hydrolyzed upon the addition of water, leading to the release of fructose. This step contributes to the overall fructose yield. Furthermore, the enhanced fructose production in ethanol can also be explained by solvation effects. [43]. Literature reports indicate that the solvation enthalpy of fructose is considerably more favorable than that of glucose. This thermodynamic preference stabilizes fructose in ethanol, thereby shifting the reaction equilibrium toward its formation. The reusability of Hf-LigS catalysts was shown in Figure 5c. The results showed that the catalytic activity could be retained after being recycled three times, indicating that the structure of coordination polymers was stable in the isomerization process. Fructose dehydration to HMF.

#### 3.3.2. Fructose Dehydration to HMF

**Figure 6:**
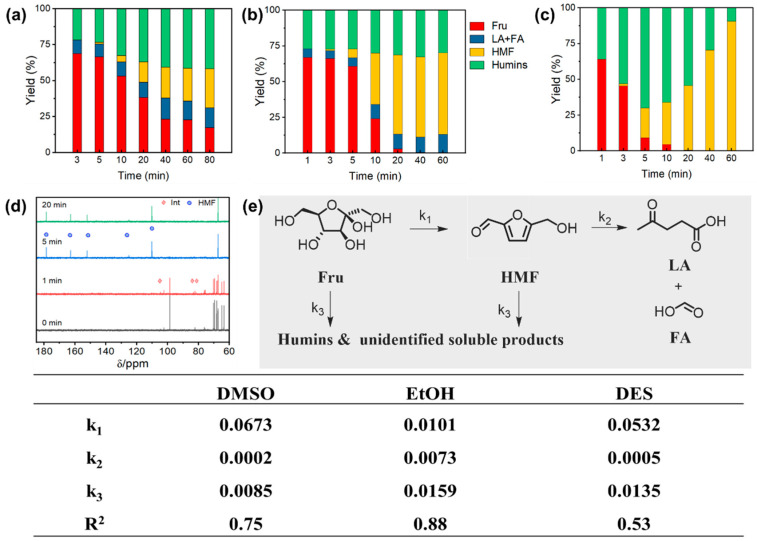
Influence of reaction solvents on fructose dehydration in (**a**) DMSO, (**b**) Ethanol, and (**c**) DES system. (**d**) ^13^C NMR spectra at different times and (**e**) catalytic constant of each reaction step.

Fructose dehydration was evaluated in three distinct solvent environments: a protic solvent (ethanol), an aprotic solvent (DMSO), and a deep eutectic solvent (DES). A marked difference in reaction rate was observed. As shown in Figure 6a,b, the formation of HMF progressed considerably more slowly in ethanol than in either DMSO or DES. In contrast, the yield of HMF increased rapidly within 10 min in the presence of DMSO and DES. Especially, more HMF was generated in the DES system at a lower reaction temperature along with decreasing content of humin as time went by (Figure 6c), achieving the highest yield of HMF (90.2%). The reaction rate of fructose dehydration to HMF in DMSO was much higher than that in ethanol. It has been reported that the pathway could be changed by tuning the solvent, and the conversion reaction in DMSO is considered to occur via the *β*-furanose form of fructose, which was conducive to dehydration to HMF. However, this phenomenon did not exist in ethanol solvent [9,44]. As presented in Figure 6d, the temporal evolution of the fructose dehydration process in a DES medium was monitored by in situ ^13^C NMR [9,44]. The initial spectrum (t = 0 min) displayed signals between 60–105 ppm, characteristic of cyclic fructose [45]. After one minute, three new weak peaks emerged at 81.46, 83.26, and 104.69 ppm, suggesting the formation of reaction intermediates. By 5 min, the spectrum became dominated by peaks at 56.5, 110.0, 124.6, 151.8, 162.0, and 178.3 ppm, which are unequivocally assigned to HMF. After 20 min, the spectral profile showed no significant changes, confirming HMF as the stable final product. Additionally, the coordination polymer retained high catalytic activity over five consecutive cycles (Figure S11), demonstrating its robust structural stability under the reaction conditions.

The reaction rate constants for each step, derived from data fitting (Figure S9), are summarized in Figure 6e. The fructose dehydration rate constant (k_1_) in DMSO and DES was six and five times higher, respectively, than that in ethanol, confirming the superiority of aprotic solvents for this step. Furthermore, the low k_2_ values in DMSO and DES indicate effective suppression of HMF rehydration to levulinic and formic acids. Conversely, ethanol exhibited the highest humin formation rate, rendering it unsuitable for processes targeting HMF. In the kinetic analysis of the DES system, intermediates that were unstable or not fully detected were treated as humin in the model. This assumption likely contributed to the moderate value of the coefficient of determination.

#### 3.3.3. Glucose Dehydration to HMF

**Figure 7:**
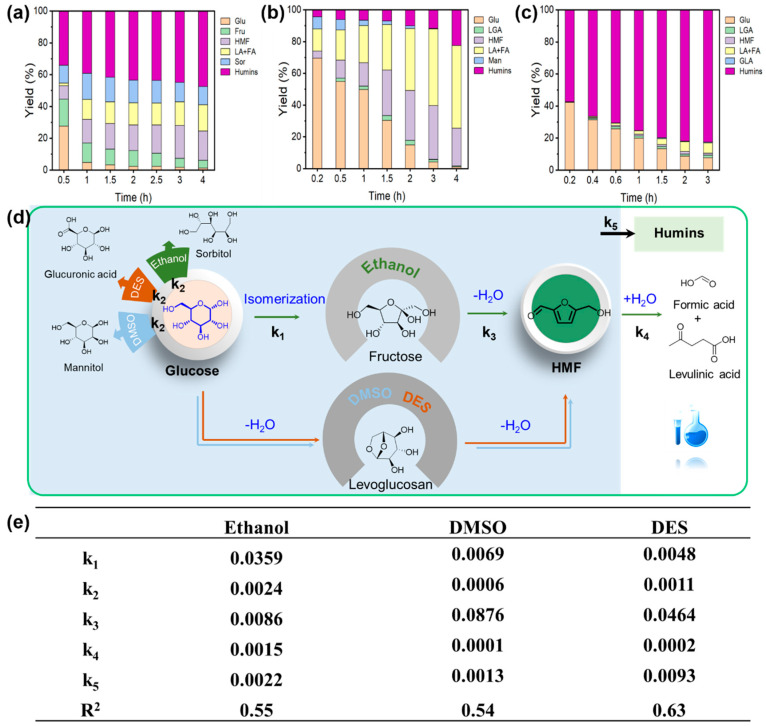
Influence of reaction solvents on glucose dehydration in (**a**) Ethanol, (**b**) DMSO, and (**c**) eutectic system. (**d**) Main reactions involved in the kinetic model of glucose dehydration in different solvents and (**e**) catalytic constant of each reaction step.

The one-pot conversion of glucose to HMF was evaluated in different solvents (Figure 7a–c). In all cases, glucose conversion increased with reaction time. The dehydration rate was highest in ethanol, achieving nearly complete conversion within 1 h, four times faster than in DMSO. However, DMSO afforded the highest HMF yield (34.0%), albeit with more significant rehydration to levulinic acid (LA) and formic acid (FA). In contrast, the deep eutectic solvent (DES) system produced only trace amounts of HMF.

Notably, the glucose dehydration pathway was strongly solvent-dependent. As illustrated in Figure 7d, in ethanol, glucose was first isomerized to fructose and then dehydrated to HMF, with sorbitol detected as a byproduct. In DMSO, however, glucose was initially dehydrated to levoglucosan (LGA), which was subsequently converted to HMF, accompanied by mannitol as a side product. In the DES system, LGA and glucaric acid (GLA) were identified as intermediates and byproducts, respectively. Across all solvents, HMF underwent rehydration to LA and FA, while sugars and derivatives polymerized to form humin. These pathway divergences can be attributed to complex solvent–solute interactions that alter the reaction thermodynamics. Significantly, despite the pronounced solvent effect on reaction pathways, the Hf-LigS catalyst demonstrated excellent structural stability and recyclability, as evidenced by its consistent catalytic performance over five consecutive cycles in DMSO (Figure S11).

A comparison between experimental and calculated values is provided in Figure S10, and the corresponding reaction rate constants for each step are summarized in Figure 7e. The isomerization of glucose to fructose (k_1_) proceeded most rapidly in ethanol. In contrast, the direct dehydration of glucose to levoglucosan (LGA) was slower in aprotic solvents (DMSO and DES). As noted earlier, the formation of ethyl fructoside in ethanol shifted the reaction equilibrium toward fructose production. The formation rate of mannitol (k_2_) was lowest in DMSO. Significant differences were observed in the dehydration rate constant (k_3_) for converting intermediates (fructose or LGA) to HMF, with DMSO exhibiting the highest value, indicating that the aprotic medium facilitates LGA dehydration. Furthermore, in DMSO and DES, k_1_ was considerably lower than k_3_, resulting in minimal LGA accumulation due to its rapid conversion to HMF. In ethanol, however, substantial fructose residue was observed, reflecting the opposite kinetic relationship. These results demonstrate that polar aprotic solvents (e.g., DMSO) enhance dehydration and suppress isomerization, favoring the LGA pathway from glucose. Conversely, polar protic solvents (e.g., ethanol) promote isomerization over dehydration, leading to fructose as the main intermediate. In addition, the higher k_4_ value in ethanol indicates a greater tendency for HMF to rehydrate to LA and FA, while the notably high k_5_ in DES suggests that this system promotes humin formation during glucose dehydration.

## 4. Conclusions

In this study, inorganic–biopolymer hybrid catalysts (Hf–LigS) were successfully synthesized via a hydrothermal self-assembly route between metal ions and renewable lignosulfonate. The inherent functional groups of lignosulfonate provided balanced Lewis acid–base pairs and Brønsted acid sites (-SO_3_H), which collectively governed the rate and pathway selectivity in hexose dehydration. We demonstrated that glucose isomerization to fructose was most effective over catalysts with moderate Lewis acid–base and Brønsted acid sites, with the latter being essential for the subsequent fructose dehydration to HMF. Solvent selection critically influenced the reaction network: DES enabled a 90.2% HMF yield from fructose, while DMSO was optimal for the one-pot glucose to HMF conversion. Notably, Hf-LigS exhibited superior performance in concentrated glucose systems (>20 wt%) in ethanol, highlighting its practical potential. This work underscores the importance of rationally integrating natural materials, solvent environments, and active sites to steer reaction selectivity, providing a sustainable strategy for biomass valorization that reduces reliance on fossil-based resources.

## Data Availability

The data that support the findings or conclusions in this study are available in the article or the Supporting Information. The details of sample characterization, including HAADF STEM images, TGA curve, and Pyridine-FTIR spectra, have been included in the Supporting Information. In addition, the other data that support the findings of this study are available from the corresponding author upon reasonable request.

## Supplementary Materials

The following supporting information can be downloaded at: https://www.mdpi.com/article/10.3390/ma18245584/s1.
