# Supplementary material for: Metal-Coordinated Lignosulfonate Catalysts for the Selective Conversion of Hexose: Active Site and Reaction Medium
Source: Materials (Basel). 2025 Dec 12;18(24):5584. doi: 10.3390/ma18245584 (PMC12735224; doi:10.3390/ma18245584)
Supplement: Supplementary file 1 [file materials-18-05584-s001.zip › materials-3985316-supplementary.pdf]

Metal-coordinated lignosulfonate catalysts for the selective conversion of hexose: Active site and reaction medium

Luyu Chen [1] , Haoyu Zhang [1] , Yirong Feng [1] , Lei Shen[4] , Lili Zhao[3] and Wei He[1,2,*]

[1] College of Biotechnology and Pharmaceutical Engineering, Nanjing Tech University, Nanjing 211816, P. R. China; hewei@njtech.edu.cn

[2] State Key Laboratory of Materials-Oriented Chemical Engineering, Nanjing Tech University, Nanjing 210009, P. R. China; hewei@njtech.edu.cn

[3] Institute of Advanced Synthesis, School of Chemistry and Molecular Engineering, Nanjing Tech University, Nanjing 211816, P. R. China; ias_llzhao@njtech

***** Correspondence: hewei@njtech.edu.cn; Tel.: +18068834182

Characterization of Hf-LigS

The crystalline structure of the catalyst was analyzed by powder X-ray diffraction analysis (XRD) using a Bruker-AXS D8 advance instrument equipped with CuKα radiation source. The morphology of the catalyst was analyzed by scanning electron microscopy (SEM) and transmission electron microscopy (TEM) using SU8010 and JEOL JEM-2100F, respectively. Specific surface areas were calculated in accordance with BET method from the nitrogen adsorption data using a Micromeritics ASAP 2460. The temperature programmed desorption was conducted by using AutoChem1 II 2920. The intensity and distribution of acidic sites was analyzed by temperature programmed desorption of ammonia (NH_3-TPD). Similarly, the intensity and distribution of basic sites was analyzed by temperature programmed desorption of carbon dioxide (CO_2-TPD). The acid type in the coordination polymer was determined through pyridine adsorption infrared spectroscopy on a Thermo fisher Nicolet iS50 equipment. Fourier infrared spectra (FT-IR) of the catalysts were recorded using Fourier infrared spectrometer (Bruker Tensor). The thermos gravimetric analysis was conducted in thermal gravimetric analyzer (TGA 550). X-ray photoelectric spectrometry was recorded in a Thermo ESCALAB 250XI equipment. The liquid was quenched with H_2SO_4 aqueous solution (5 mM) at room temperature for 1 hour and subsequently quantified by high performance liquid chromatography (HPLC, Agilent 1260) assembled with a DAD detector and Aminex HPX-87H. The mobile phase was H_2SO_4 aqueous solution (0.005 M) at a flowrate of 0.6 mL/min. The peaks for different compounds were confirmed and quantified using external standards.

Catalyst characterization

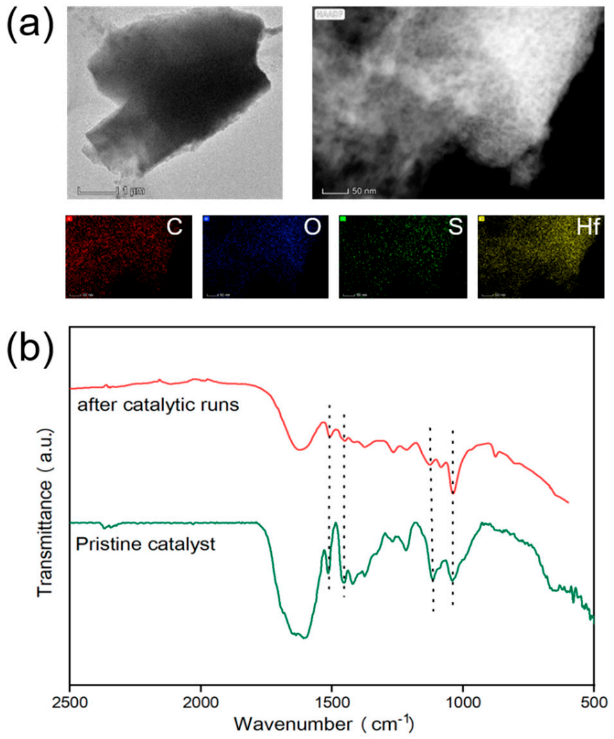

Figure S1. (a)TEM diagram of Hf-LigS, scanning transmission electron microscopy of high-angle annular dark-field (HAADF STEM) image and corresponding elemental mapping of C, O, S and Hf. (b)FT-IR spectroscopic comparison of Hf-LigS before and after the reaction at 170°C.

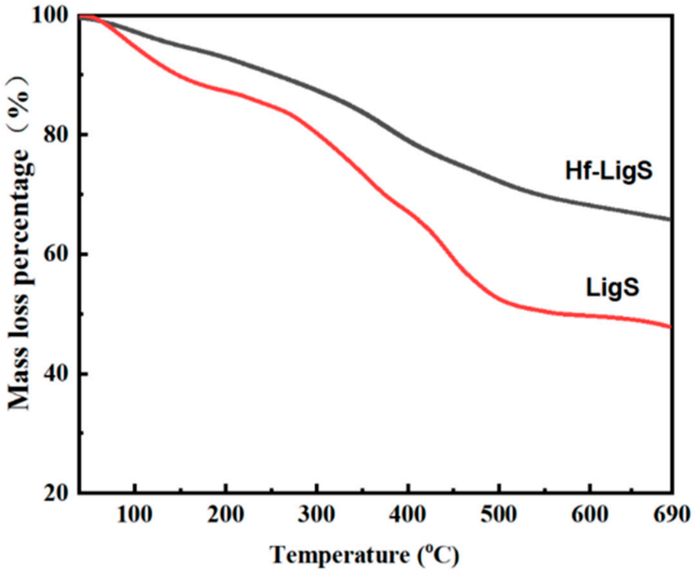

Figure S2. TGA curve of Hf-LigS and LigS.

Nature and content of acid and base sites

Table S1 The acidity and basicity ratio of different catalysts

Sample	Acidity (μmol NH_3/g)	Basicity (μmol CO_2/g)	Acid/Base ratio
Hf-LigS	1865.48	777.89	2.40
Ru-LigS	3663.68	703.78	5.21
Zr-LigS	1948.66	994.85	1.96
Fe-LigS	2276.98	1087.96	2.09
Acid-LigS	250.14	/	-
Hf-Lig	7644.76	2060.88	3.71

The bands located at 1459, 1540, 1620 and 1488 cm^{-1} were attributed to the pyridine interacting with Lewis acid sites (LA), Brønsted acid sites (BA), LA and LA+BA, respectively.

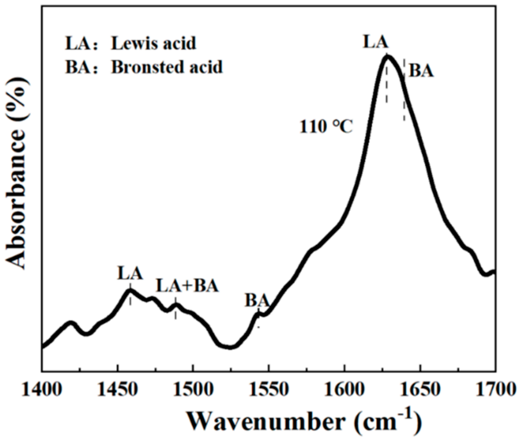

Figure S3. Pyridine-FTIR spectra of Hf-LigS at 110 ºC.

Glucose isomerization

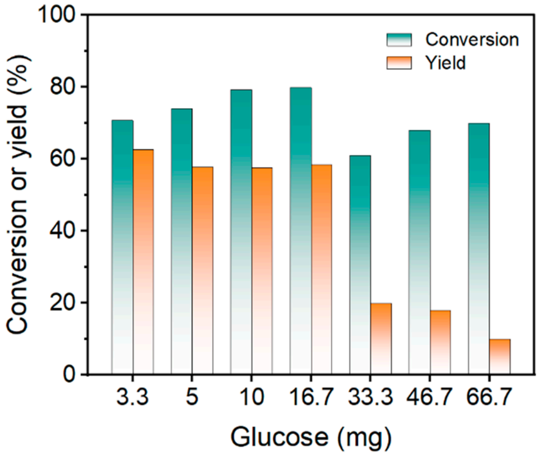

Figure S4. The effect of glucose concentration on the glucose isomerization. Reaction conditions: Ethanol 1 mL, 100 °C, 18 h.

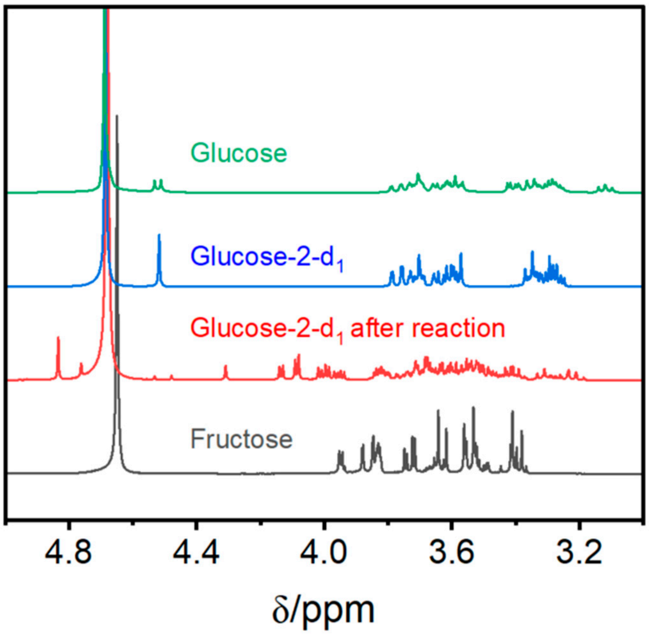

Figure S5. ^{1}H NMR spectra of glucose-2-d1 before and after the reaction in ethanol

Kinetic modeling

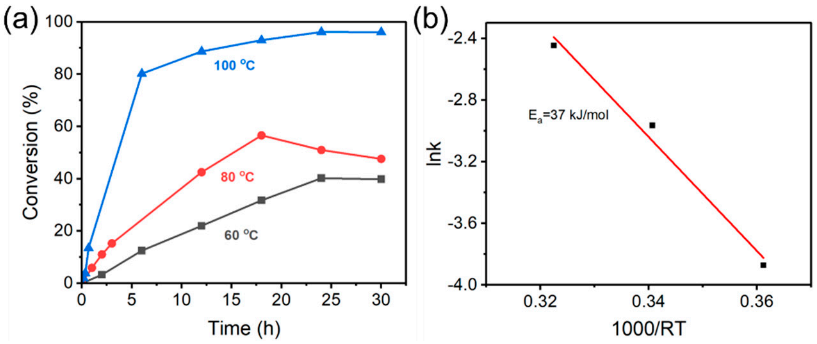

Figure S6. (a) Effect of temperature and time on glucose conversion, (b) Relationship between lnk and 1000/T.

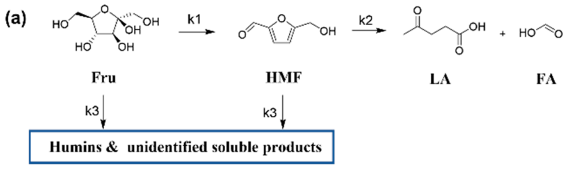

$$r_{Fru} = \frac{d[Fru]}{dt} = -k_1 \cdot [Fru] - k_3 \cdot [Fru] \qquad r_{FA+LA} = \frac{d[FA+LA]}{dt} = k_2 \cdot [HMF]$$

$$r_{HMF} = \frac{d[HMF]}{dt} = k_1 \cdot [Fru] - k_2 \cdot [HMF] - k_4 \cdot [HMF] \qquad r_{Hum} = \frac{d[Hum]}{dt} = k_3 \cdot [Fru] + k_3 \cdot [HMF]$$

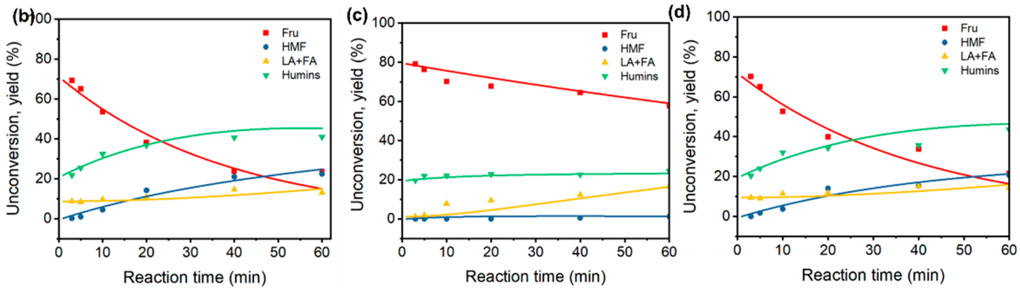

Figure S7 (a) Main reactions of fructose dehydration involved in kinetic model, experimental versus calculated unconversion and yields for Hf-LigS(b), Hf-Lig(c), Acid-LigS(d)

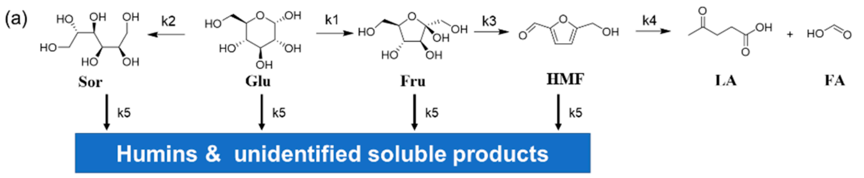

$$r_{Glu} = \frac{d[Glu]}{dt} = -k_1[Glu] - k_2[Glu] - k_5[Glu] \qquad r_{FA+LA} = \frac{d[FA+LA]}{dt} = k_4[HMF]$$

$$r_{Fru} = \frac{d[Fru]}{dt} = k_1[Glu] - k_3[Fru] - k_5[Fru] \qquad r_{Sor} = \frac{d[Sor]}{dt} = k_2[Glu] - k_5[Sor]$$

$$r_{HMF} = \frac{d[HMF]}{dt} = k_3[Fru] - k_4[HMF] - k_5[HMF] \qquad r_{Hum} = \frac{d[Hum]}{dt} = k_5[Glu] + k_5[Flu] + k_5[HMF] + k_5[Sor]$$

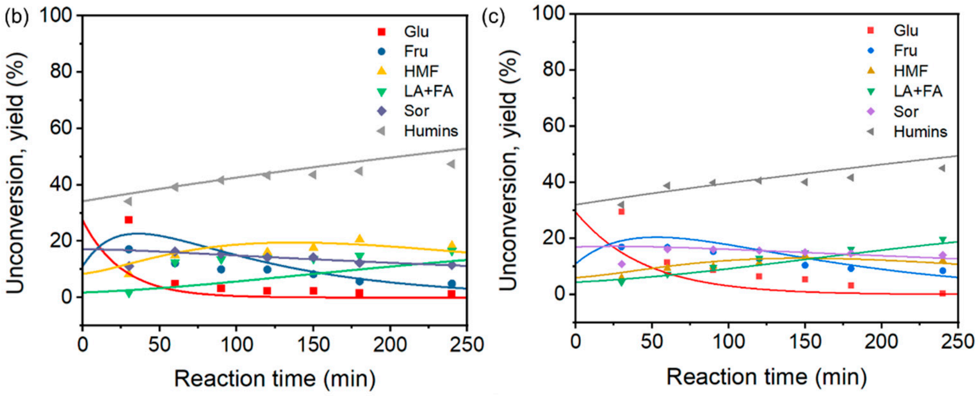

Figure S8 (a) Main reactions of glucose dehydration involved in kinetic model, experimental versus calculated unconversion and yields for Hf-LigS(b), D. Hf-Lig(c).

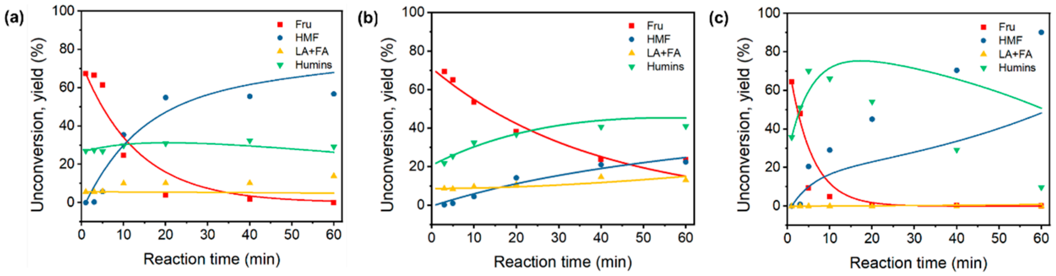

Figure S9 Experimental versus calculated unconversion and yields of fructose dehydration under (a) DMSO, (b) ethanol, (c) DES.

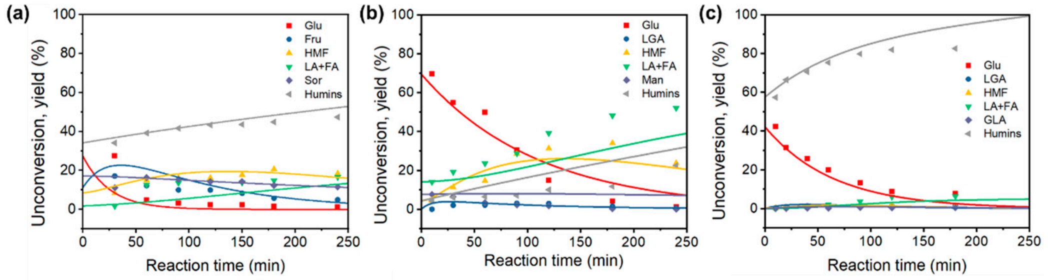

Figure S10. Experimental versus calculated unconversion and yields of glucose dehydration under ethanol (a), DMSO (b), and DES(c).

Reusability of Hf-LigS

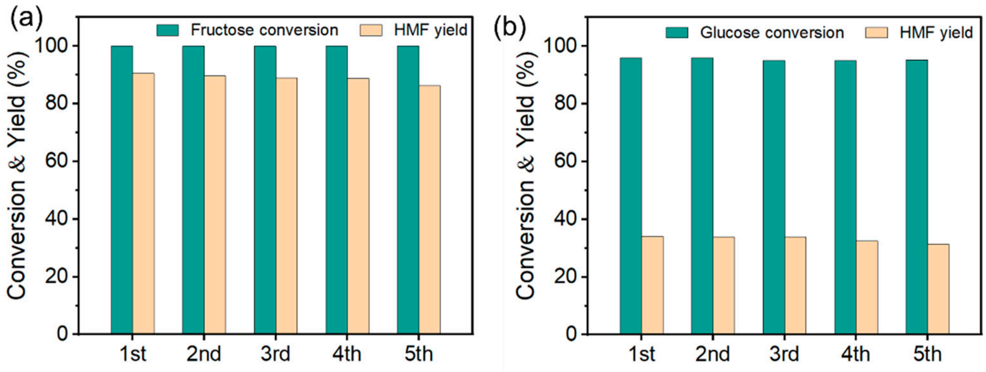

Figure S11. Reusability of Hf-LigS in fructose dehydration reaction, DES solvent, time=1 h(a), and glucose dehydration reaction, DMSO solvent, time=3 h(b).

Computational Details

Geometry optimizations were carried out using the Gaussian 16 program with the RBP86 functional and the def2-SVP basis set and dispersion corrections by Grimme with Becke-Johnson damping (D3BJ), which is termed as RBP86+D3BJ/def2-SVP. Frequency results were examined to confirm the stationary points as transition states (only one imaginary frequency) or minima (no imaginary frequencies) and used to obtain the zero-point energy (ZPE)-corrected enthalpies and free energies at 298.15 K and 1 atm. Intrinsic reaction coordinate analysis was performed to evaluate the correct connections between the transition states and corresponding minima if necessary.

Note that the catalyst was simplified duo to the complex structure of this coordination polymer. Meanwhile, the coordination between Hf^{4+} and phenolic hydroxyl group was retained to construct the Lewis base- and Lewis acid sites. The energetics was further improved by using the larger basis set def2-TZVPP, denoted as the RBP86+D3BJ/def2-TZVPP(SMD, solvent=Ethanol)//RBP86+D3BJ/def2-SVP(SMD, solvent=Ethanol)

level; electronic energies without ZPE corrections are also given for reference in the related schemes.

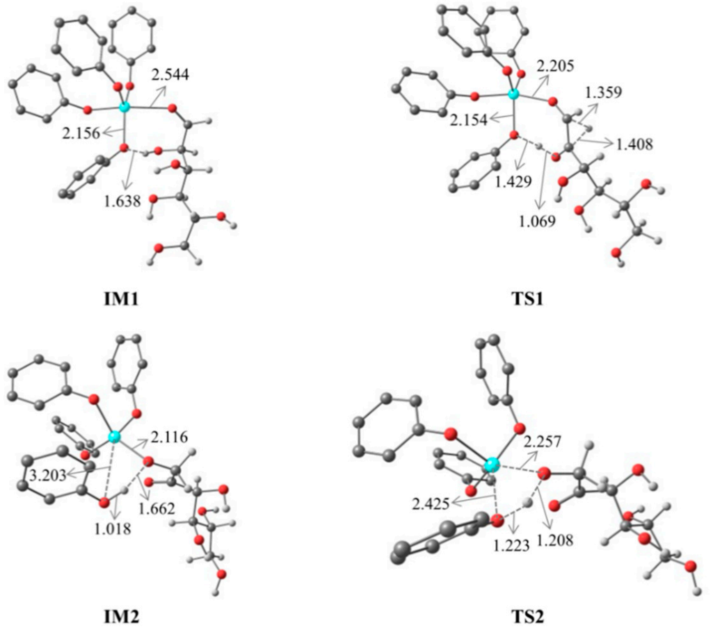

Figure S12. Optimized structures of some key stationary points according to Figure. 6 at the RBP86+D3BJ/def2-TZVPP(SMD, solvent=Ethanol)//RBP86+D3BJ/def2-SVP(SMD, solvent=Ethanol) level. Key bond distances are given in Å. Trivial hydrogen atoms have been omitted for clarity. Color code: Hf, blue; O, red; C, gray; H, white.

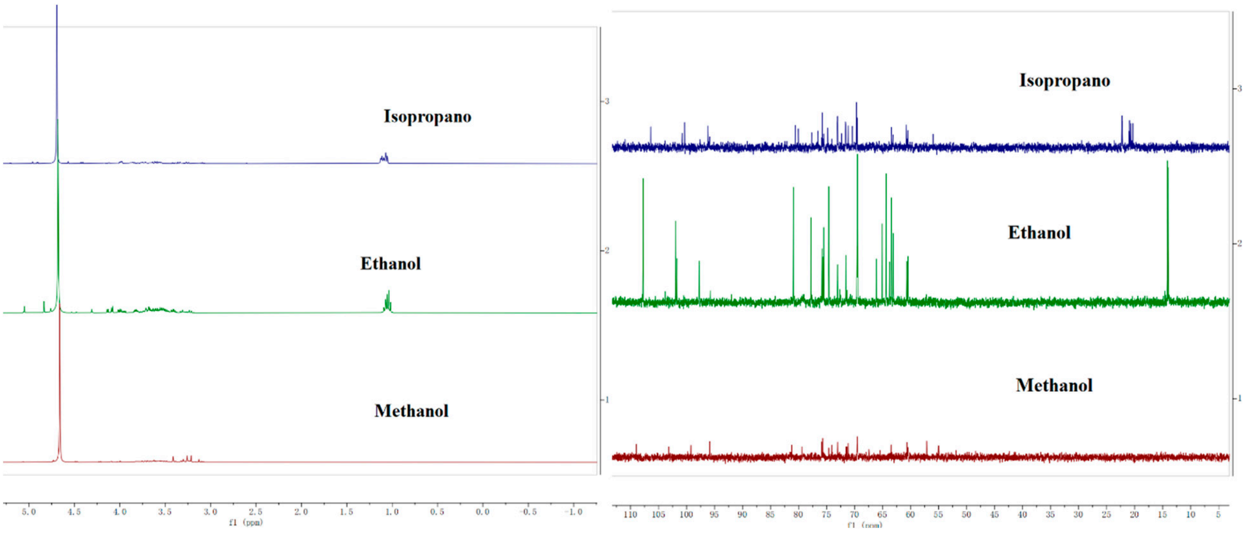

Figure. S13. ^{1}H NMR and ^{13}C NMR spectra in alcohol solvents.

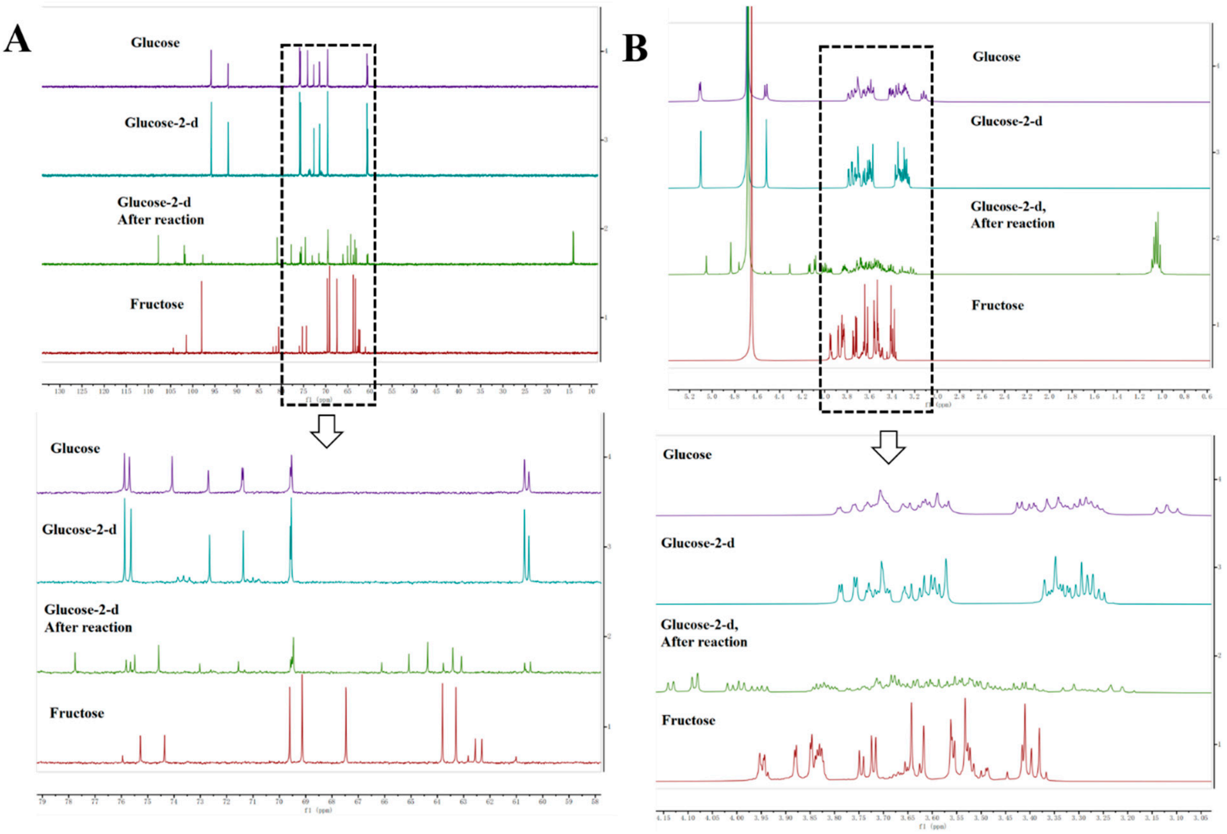

Figure S14. NMR spectra of glucose-2-d_1 before and after the reaction in ethanol.

(A) ^{13}C NMR spectra. (B) ^{1}H NMR spectra.

When glucose-2-d_1 was used as the raw material, the reaction media ethanol was evaporated under vacuum and analyzed by ^{1}H NMR and ^{13}C NMR. The difference of ^{13}C NMR results (Figure S14A) between reaction mixture before hydrolysis (δ/ppm=107.8, 101.9, 101.7, 97.7) and fructose (δ/ppm=104.4, 101.4, 98.0) revealed that the presence of characteristic peaks of ethyl fructoside [11]. The presence of characteristic peaks (δ/ppm=14.0-14.2) revealed that C atoms in alkyl groups existed in the reaction mixture [37]. The existence of characteristic peaks in alkyl groups based on the ^{1}H NMR results (δ/ppm=1.02-1.09) also confirmed that resulting fructose reacted with ethanol, affording ethyl fructoside (Figure S14B). When MeOH and isopropanol were used as the solvent, similar characteristic peaks of alkyl groups in ^{1}H NMR and ^{13}C NMR spectra were also observed (Figure S13). This phenomenon demonstrated that the formation of alkyl fructoside catalyzed by Lewis acid sites occurred in all three alcohol solvents.

Table S2. Turnover Frequency (TOF) Value for the Initial Glucose Isomerization Reaction.

Reaction	Conditions	TOF (h^{-1})

Glucose Isomerization	100 °C, Ethanol, 18 h	3.1
Fructose Dehydration to HMF	140 °C, DMSO, 1 h	436.0
Fructose Dehydration to HMF	120 °C, DES, 1 h	206.1
One-pot Glucose to HMF	170 °C, DMSO, 3 h	68.7

Table S3. Comparison of Hf-LigS with Other Advanced Catalysts in Glucose Isomerization and HMF Production.

catalyst	Temperature/°C	solvent	fructose yield /%	HMF yield/%
Sn-β	90	Water/Dioxane	41.5	56
MOF-808	160	DMSO	37	31
Hf–LigS	100/120	Ethanol/EDS	58.4	90.2

Table S4. Coordinates and energies (in hatree) of the calculated structures at the RBP86+D3BJ/def2-TZVPP (SMD, solvent=Ethanol)//RBP86+D3BJ/def2-SVP (SMD, solvent=Ethanol) level.

Glucose

Free energy：-687.383075

Energy：-687.529339

C	0.72668000	0.39123400	0.17876000
C	1.61489000	-0.73561700	-0.37460700
C	-2.95205000	-0.58162000	-0.62565600
C	-1.69154000	-0.50016100	0.22105200
C	-0.67755000	0.44022900	-0.46569100
H	1.72413300	-0.56719100	-1.47445700
H	0.60187400	0.23964900	1.28008700
H	-2.78128100	-0.95702500	-1.67886500
H	-0.58408700	0.17290300	-1.54728200
O	-1.22121500	1.75195700	-0.31342900

H	-0.43036000	2.34327300	-0.31122000
O	1.31864000	1.66256100	-0.09103800
H	2.30766300	1.52913500	-0.05479800
O	-1.95515400	-0.07705900	1.54236600
H	-2.04051200	0.90169200	1.46566000
O	-4.06636500	-0.28041800	-0.22862700
O	0.98150900	-1.98147100	-0.09781100
C	3.01338000	-0.72545300	0.26104100
H	3.58489000	-1.60887600	-0.10954700
H	2.90796800	-0.82292000	1.36539000
O	3.68045100	0.49614700	-0.09775500
H	4.39692400	0.65818300	0.54855900
H	1.45210200	-2.67421500	-0.60384300
H	-1.24510800	-1.52002200	0.24128400

Hf-catalyst

Free energy：-1276.227052

Energy：-1276.533784

C	-6.06378700	-0.79685200	-0.12968300
C	-4.86162900	-1.52296200	-0.10309200
C	-3.67097100	-0.92158100	0.37784100
C	-3.72598500	0.41985400	0.84227500
C	-4.93212900	1.13793700	0.80441100
C	-6.10948300	0.53730100	0.31860700
H	-6.97811000	-1.28093000	-0.51108500
H	-4.82008800	-2.56461300	-0.46030300
H	-2.80861000	0.88782300	1.23697000
H	-4.95168200	2.17985100	1.16434200
H	-7.05412500	1.10290500	0.29173700
O	-2.51409400	-1.62136700	0.39024500
Hf	-0.64240100	-0.83693600	-0.10556000

O	-0.80442100	0.78405700	-1.36589100
C	-0.04491700	1.90383800	-1.37190600
C	1.34757800	1.83921900	-1.62988200
C	-0.63678600	3.16548600	-1.12045800
C	2.12468000	3.00752600	-1.62511300
H	1.80943200	0.85815300	-1.82290700
C	0.15062000	4.32936100	-1.12091300
H	-1.71866900	3.21126100	-0.91645500
C	1.53378500	4.26031000	-1.37117400
H	3.20796100	2.93578000	-1.81455100
H	-0.32463200	5.30315800	-0.91738500
H	2.14791700	5.17442500	-1.36340500
O	0.30142800	-0.11052300	1.55638700
O	0.59011700	-2.23415600	-0.99559200
C	1.32691600	0.77905700	1.61473800
C	1.06848200	2.14537100	1.88241000
C	2.66449700	0.35512200	1.42513700
C	2.12548900	3.06663900	1.93903600
H	0.02668000	2.47037900	2.03073800
C	3.71414300	1.28753900	1.48055000
H	2.86588300	-0.70884700	1.22828100
C	3.45427900	2.64656200	1.73526600
H	1.90655500	4.12864500	2.13568300
H	4.74862900	0.94208000	1.32050900
H	4.27992500	3.37444100	1.77380900
C	1.89623000	-2.44460600	-0.71948400
C	2.28363600	-3.21774900	0.40429200
C	2.90499900	-1.87760800	-1.53845300
C	3.64286600	-3.38772200	0.71436600
H	1.49899500	-3.66336400	1.03711800

C	4.26126600	-2.05158700	-1.21751300
H	2.60354000	-1.28802600	-2.41907000
C	4.64072000	-2.80141000	-0.08799500
H	3.92541800	-3.98076900	1.59966800
H	5.03145800	-1.59020800	-1.85722000
H	5.70513600	-2.93113800	0.16308300

IM1

Free energy：-1963.598711

Energy：-1964.076730

C	-2.16987300	3.95203800	-1.25109200
C	-2.04021200	2.56123700	-1.39743800
C	-1.59685900	1.76890600	-0.31077300
C	-1.30682700	2.40012100	0.92519700
C	-1.43638600	3.79202600	1.05923300
C	-1.86417400	4.57861300	-0.02711600
H	-2.50821400	4.55648700	-2.10880700
H	-2.26926600	2.06584400	-2.35397100
H	-0.96793900	1.78215100	1.77193100
H	-1.19586200	4.26738700	2.02437700
H	-1.96092500	5.67052900	0.07925200
O	-1.44072500	0.43508600	-0.45575300
Hf	0.42450100	-0.61142500	-0.18530200
O	1.25383700	0.88779100	-1.27448300
C	2.18601900	1.85402600	-1.15415400
C	3.56329700	1.54409200	-1.27780000
C	1.80092400	3.19554900	-0.91406500
C	4.53002900	2.55115700	-1.13840400
H	3.85648800	0.49904300	-1.46106700
C	2.77899600	4.19460700	-0.77516900

H	0.73015300	3.43390000	-0.82447000
C	4.14725800	3.88228600	-0.88184200
H	5.59752600	2.28945800	-1.22142300
H	2.46372900	5.23237600	-0.57696400
H	4.90971500	4.66871600	-0.76633200
O	1.41148500	-0.05915000	1.50641300
O	1.35407200	-2.05238300	-1.37266700
C	2.67052700	0.37613000	1.75415200
C	2.88639100	1.71252200	2.16810500
C	3.78097300	-0.48923100	1.60541800
C	4.19068100	2.17622100	2.39877600
H	2.01915800	2.38192200	2.27785200
C	5.08175100	-0.01279600	1.83742500
H	3.61104900	-1.53080400	1.29159900
C	5.29722700	1.32076000	2.23119100
H	4.34530600	3.22430000	2.70266600
H	5.93619300	-0.69619500	1.70312200
H	6.31934400	1.69226400	2.40523100
C	2.50510300	-2.67166300	-1.03533200
C	2.54599500	-3.62548200	0.01542400
C	3.70848500	-2.36636100	-1.72270300
C	3.76107300	-4.22720800	0.38274000
H	1.61162100	-3.87414200	0.54353300
C	4.91729500	-2.97307800	-1.34567900
H	3.67382000	-1.63727400	-2.54775800
C	4.95556900	-3.90237500	-0.28818800
H	3.77392600	-4.95772000	1.20842600
H	5.84325700	-2.71345900	-1.88487900
H	5.90652100	-4.37364200	0.00588000
C	-5.15434000	-0.90719900	-0.18991600

C	-6.34967000	-0.63142200	0.74485000
C	-2.03029700	-3.05271000	0.50316100
C	-3.08815000	-2.44751800	-0.38566400
C	-4.06321700	-1.69244500	0.57750000
H	-5.94673200	-0.19867700	1.69186800
H	-5.52835500	-1.53131000	-1.03779100
H	-2.37208000	-3.89162700	1.16503700
H	-4.56666300	-2.45614200	1.20667600
O	-3.36936200	-0.80414000	1.44421800
H	-3.22146400	0.00395600	0.89417700
O	-4.56592200	0.29898500	-0.66464800
H	-5.21810500	1.03332100	-0.48540600
O	-2.58176100	-1.69030000	-1.45465600
H	-2.31441400	-0.77855500	-1.12464000
O	-0.85919900	-2.67422300	0.56776100
O	-6.99168600	-1.88207000	0.97244700
C	-7.34779800	0.37088300	0.15024200
H	-8.24849200	0.41398100	0.80598500
H	-7.67275100	0.02353100	-0.85739100
O	-6.70403000	1.65525700	0.08377600
H	-7.20145400	2.22000700	-0.54182400
H	-7.51455700	-1.80125900	1.79469600
H	-3.66702800	-3.30473700	-0.80555400

TS1

Free energy：-1963.570502

Energy：-1964.043601

C	-0.50181800	3.72642000	-2.54963500
C	-0.39379900	2.32721700	-2.50574800
C	-1.03302100	1.59974300	-1.47715900

C	-1.80681300	2.28953900	-0.51499400
C	-1.91822000	3.68951300	-0.57644100
C	-1.26146400	4.41680300	-1.58585900
H	0.01615600	4.28439500	-3.34661200
H	0.20424800	1.78098900	-3.25149800
H	-2.31823700	1.72632000	0.28133800
H	-2.51927900	4.21638400	0.18260300
H	-1.34137200	5.51463100	-1.62284500
O	-0.89584500	0.24115200	-1.40986200
Hf	0.68491800	-0.79041700	-0.37187900
O	1.86189400	0.80429600	-0.83504900
C	2.47701900	1.88931700	-0.33902600
C	3.89032200	1.98582100	-0.39238400
C	1.73951000	2.94928200	0.24576200
C	4.54475100	3.09923600	0.15563800
H	4.46014900	1.15769400	-0.84180000
C	2.40582400	4.06186500	0.78298600
H	0.64255400	2.87625900	0.28470100
C	3.81054000	4.14484400	0.74921300
H	5.64572900	3.14742100	0.12453500
H	1.81565500	4.87270900	1.24120300
H	4.32940900	5.01557700	1.18025400
O	1.30254400	-0.49380500	1.58939900
O	1.66318200	-2.30896200	-1.39057600
C	2.52421600	-0.14291200	2.04380000
C	2.67584700	1.00744600	2.85934400
C	3.67876000	-0.90017100	1.71954300
C	3.94770100	1.40980100	3.29486100
H	1.77948800	1.59293600	3.11709800
C	4.94797700	-0.48736500	2.15769400

H	3.57062500	-1.80737100	1.10550200
C	5.09484200	0.67216700	2.94116300
H	4.04503300	2.31944000	3.90989800
H	5.83260500	-1.08254000	1.87724700
H	6.09233300	0.99773700	3.27609700
C	2.97382300	-2.63235100	-1.33645300
C	3.39551900	-3.79227100	-0.63898800
C	3.95269800	-1.81588700	-1.95827700
C	4.76153700	-4.10551900	-0.54174800
H	2.63273800	-4.42555500	-0.15790300
C	5.31549700	-2.13594200	-1.85032700
H	3.62547400	-0.91835200	-2.50610800
C	5.73148600	-3.27793700	-1.13891700
H	5.07170200	-5.00499400	0.01525000
H	6.06328700	-1.48099000	-2.32717400
H	6.80194600	-3.52256500	-1.05389700
C	-5.87448200	-0.81044100	0.16850400
C	-7.10572200	-0.92763800	1.08843700
C	-2.22724700	-1.54778900	1.05327300
C	-3.31297400	-0.97970000	0.28793400
C	-4.59740800	-0.59607700	1.01700200
H	-7.10814000	-0.03964700	1.76570000
H	-5.78459700	-1.76700700	-0.40152700
H	-2.44869600	-1.71764100	2.13214200
H	-4.67860500	-1.20384500	1.94182400
O	-4.49709600	0.77927800	1.36394800
H	-4.93297200	1.22500100	0.58904000
O	-5.97854000	0.30818700	-0.70914600
H	-6.95248200	0.52585300	-0.77540400
O	-3.18742000	-0.61881700	-0.96976500

H	-2.17496000	-0.35756900	-1.19045600
O	-1.03381900	-1.76564800	0.60588400
O	-6.96222700	-2.14147100	1.81827600
C	-8.42364200	-0.94512100	0.30337100
H	-9.26341600	-1.14306800	1.00951700
H	-8.39762500	-1.77396400	-0.44039200
O	-8.58876400	0.33189300	-0.33761800
H	-9.22089600	0.22573800	-1.07711000
H	-7.54431700	-2.08640800	2.60230400
H	-3.19645400	-2.35774400	0.55246400

IM2

Free energy：-1963.609393

Energy：-1964.089701

C	2.11777500	4.82282500	-1.33414100
C	0.89096600	4.16398300	-1.50918300
C	0.19607900	3.65459500	-0.38739000
C	0.75363400	3.81049300	0.90527800
C	1.98528600	4.46850100	1.06393900
C	2.67561500	4.97937100	-0.04986300
H	2.64971600	5.21372700	-2.21672900
H	0.45232100	4.03103600	-2.51064000
H	0.20274500	3.42709400	1.77828900
H	2.40821800	4.57979200	2.07552100
H	3.64250400	5.48981400	0.07918500
O	-0.98930700	3.01886300	-0.58922200
Hf	0.48314600	0.39610900	0.51310300
O	2.38227200	0.60849600	1.31910800
C	3.52444200	0.49878600	0.59737400
C	4.33824100	-0.65579500	0.71468400

C	3.91706200	1.52575300	-0.29814400
C	5.49678100	-0.78626300	-0.06856900
H	4.03531100	-1.45187400	1.41162300
C	5.08025400	1.38562300	-1.07210500
H	3.28868900	2.42548200	-0.38621000
C	5.87562900	0.22789100	-0.96897500
H	6.10779000	-1.69924300	0.02364000
H	5.36281500	2.19085100	-1.77009100
H	6.78311100	0.11802800	-1.58339200
O	0.34037000	-1.53651700	1.13115400
O	0.73966400	0.22621100	-1.54000900
C	1.31659200	-2.42609200	1.43673700
C	1.82094700	-2.50768700	2.75686700
C	1.85027800	-3.27673700	0.43921200
C	2.85873200	-3.40395000	3.06116400
H	1.39899900	-1.84460000	3.52886500
C	2.88935300	-4.16701800	0.75498300
H	1.44994000	-3.21523300	-0.58372800
C	3.40423800	-4.23487500	2.06332000
H	3.25005000	-3.44902200	4.09076300
H	3.30430300	-4.81126400	-0.03734400
H	4.22293800	-4.93081800	2.30502300
C	1.13709900	-0.90128900	-2.17796400
C	0.18703600	-1.88134000	-2.56272800
C	2.50777800	-1.12688000	-2.45946000
C	0.60682800	-3.06498400	-3.19147500
H	-0.87710600	-1.70457300	-2.33988900
C	2.91514900	-2.31650900	-3.08495100
H	3.24421800	-0.36436300	-2.16283200
C	1.97203500	-3.29491600	-3.45234800

H	-0.14321800	-3.82199700	-3.47398900
H	3.98742000	-2.48084500	-3.28115600
H	2.29681100	-4.22811100	-3.93889500
C	-5.39495900	-0.22279200	-0.24414400
C	-6.62780400	-1.13992900	-0.33524100
C	-2.43696000	0.74689500	1.72669600
C	-3.03103600	-0.09672200	0.61653100
C	-4.41574400	-0.70299800	0.85352100
H	-7.07290200	-1.21703500	0.68530300
H	-4.86619100	-0.25720300	-1.22727300
H	-3.19357100	1.53505400	1.96753600
H	-4.29912500	-1.80468400	0.75006700
O	-4.94785600	-0.40104300	2.13141900
H	-5.43921800	0.44368000	1.97208300
O	-5.75047300	1.10940400	0.11954100
H	-6.69837500	1.23642700	-0.17231200
O	-2.43189800	-0.30290200	-0.44044900
H	-1.23435200	2.49387700	0.24749600
O	-1.19893900	1.30992200	1.41403100
O	-6.17200800	-2.40461800	-0.80422900
C	-7.69035000	-0.58545200	-1.29431600
H	-8.50953200	-1.33426700	-1.39984500
H	-7.23214000	-0.43263400	-2.29809200
O	-8.19516700	0.64835200	-0.75350100
H	-8.61724300	1.15345900	-1.47769700
H	-6.83092000	-3.07556400	-0.53709700
H	-2.39961800	0.10091100	2.63982000

TS2

Free energy：-1963.598527

Energy：-1964.071406

C	2.25649900	5.15140100	-1.29661200
C	1.43069900	4.02156200	-1.41127100
C	0.50970900	3.70693700	-0.38356400
C	0.42429200	4.54810000	0.75114800
C	1.25478700	5.67684500	0.85287300
C	2.17646500	5.98579100	-0.16516200
H	2.97106400	5.38238600	-2.10357100
H	1.48506800	3.36494600	-2.29423700
H	-0.29343900	4.29982500	1.54942000
H	1.18032500	6.32255800	1.74327000
H	2.82580200	6.87109400	-0.07904000
O	-0.26993600	2.60104800	-0.50750300
Hf	0.58523700	0.43772800	0.17726200
O	2.43293500	0.91246400	0.94566100
C	3.59873400	0.52059700	0.35869700
C	4.24127100	-0.67337500	0.76651800
C	4.16712800	1.29072000	-0.68483300
C	5.41193000	-1.09901300	0.11724800
H	3.79754800	-1.26748700	1.58006700
C	5.33818900	0.85463700	-1.32584100
H	3.66366700	2.22171000	-0.99122600
C	5.96508700	-0.34446900	-0.93456500
H	5.89146700	-2.03936800	0.43457500
H	5.76225100	1.45721900	-2.14580200
H	6.87900800	-0.68652800	-1.44524300
O	0.11803500	-1.27352900	1.20385800
O	0.87014200	-0.22623000	-1.74636700
C	1.02440400	-2.12099100	1.75649900
C	1.49974700	-1.90127900	3.07271800

C	1.52779900	-3.21887700	1.01685200
C	2.47881600	-2.74611200	3.62075600
H	1.10446400	-1.04599100	3.64385500
C	2.50863000	-4.05517600	1.57471800
H	1.15263000	-3.39085100	-0.00316600
C	2.99536700	-3.82373100	2.87505400
H	2.84915100	-2.55433200	4.64124200
H	2.90172700	-4.89438500	0.97778000
H	3.76974900	-4.47806600	3.30545300
C	1.19238000	-1.49290000	-2.10342400
C	0.17531200	-2.45403600	-2.32809000
C	2.54863900	-1.87874100	-2.23909000
C	0.51493200	-3.77691100	-2.65531300
H	-0.87727400	-2.14747500	-2.21784600
C	2.87518000	-3.20581800	-2.56294100
H	3.33597900	-1.12829100	-2.06739300
C	1.86469000	-4.16414400	-2.76921500
H	-0.28620900	-4.51727300	-2.81461600
H	3.93584800	-3.49355900	-2.64837300
H	2.12594400	-5.20464000	-3.01848100
C	-5.38647600	-0.14136900	-0.29572100
C	-6.57782200	-1.11247400	-0.38806700
C	-2.39330200	0.81478200	1.53842900
C	-2.95986700	0.01581300	0.38257600
C	-4.28455200	-0.70639000	0.63337600
H	-6.89976300	-1.35557600	0.65259500
H	-4.95977500	-0.01687100	-1.32039300
H	-3.15399300	1.59071400	1.79874900
H	-4.12100900	-1.77273900	0.36249100
O	-4.71530700	-0.61851400	1.98041000

H	-5.25835700	0.20923100	1.98790400
O	-5.76464200	1.10847500	0.27671000
H	-6.74562300	1.21007700	0.10975100
O	-2.39171200	-0.05857100	-0.70802400
H	-0.96665800	2.24273500	0.43139600
O	-1.14326600	1.39248600	1.27119800
O	-6.11833700	-2.26954500	-1.07759600
C	-7.76921600	-0.49984700	-1.13709100
H	-8.55676000	-1.27902600	-1.26153700
H	-7.43952900	-0.17820400	-2.15134800
O	-8.26819600	0.60934400	-0.36926700
H	-8.80346400	1.17725700	-0.95954400
H	-6.72216400	-3.00626300	-0.85653900
H	-2.33920100	0.14211800	2.42433400

Frucose

Free energy：-687.394998

Energy：-687.541258

C	0.60801500	0.04035600	-0.28497100
C	1.94951800	0.55382100	0.26778400
C	-2.97870600	-0.49510200	0.37495400
C	-1.88338900	0.38993400	-0.17680500
C	-0.57567600	0.50401300	0.59830400
H	2.01739300	0.24702000	1.33880800
H	0.47105700	0.46396900	-1.30944600
H	-2.53959000	-1.52036500	0.47863100
H	-0.42913100	1.58364700	0.82502900
O	-0.58996500	-0.23794400	1.80452800
H	-0.25416900	-1.12574700	1.52302200
O	0.54658500	-1.38375900	-0.28848700
H	1.49218700	-1.70525300	-0.33737200

O	-2.06770700	0.98392600	-1.24145700
H	-3.83761800	0.10884600	-1.21132600
O	-4.11044200	-0.47422800	-0.45869500
O	1.94117600	1.97037000	0.12837300
C	3.14899500	-0.03362400	-0.48890100
H	4.08310300	0.44704000	-0.11552400
H	3.04923600	0.20455300	-1.57251800
O	3.18272700	-1.45382000	-0.26382700
H	3.72093700	-1.86498900	-0.97022800
H	2.60442800	2.33305300	0.74836400
H	-3.20936000	-0.16451700	1.41689200
